# Increased macroH2A1.1 Expression Correlates with Poor Survival of Triple-Negative Breast Cancer Patients

**DOI:** 10.1371/journal.pone.0098930

**Published:** 2014-06-09

**Authors:** Anne-Claire Lavigne, Magali Castells, Jérôme Mermet, Silvia Kocanova, Mathieu Dalvai, Kerstin Bystricky

**Affiliations:** 1 Université de Toulouse; Laboratoire de Biologie Moléculaire Eucaryote (LBME); F-31062 Toulouse, France; 2 CNRS; LBME; F-31062 Toulouse, France; Princess Margaret Cancer Centre, Canada

## Abstract

**Purpose:**

Epithelial-Mesenchymal Transition (EMT) features appear to be key events in development and progression of breast cancer. Epigenetic modifications contribute to the establishment and maintenance of cancer subclasses, as well as to the EMT process. Whether histone variants contribute to these transformations is not known. We investigated the relative expression levels of histone macroH2A1 splice variants and correlated it with breast cancer status/prognosis/types.

**Methods:**

To detect differential expression of macroH2A1 variant mRNAs in breast cancer cells and tumor samples, we used the following databases: GEO, EMBL-EBI and publisher databases (may-august 2012). We extracted macroH2A1.1/macroH2A1 mRNA ratios and performed correlation studies on intrinsic molecular subclasses of breast cancer and on molecular characteristics of EMT. Associations between molecular and survival data were determined.

**Results:**

We found increased macroH2A1.1/macroH2A1 mRNA ratios to be associated with the claudin-low intrinsic subtype in breast cancer cell lines. At the molecular level this association translates into a positive correlation between macroH2A1 ratios and molecular characteristics of the EMT process. Moreover, untreated Triple Negative Breast Cancers presenting a high macroH2A1.1 mRNA ratio exhibit a poor outcome.

**Conclusion:**

These results provide first evidence that macroH2A1.1 could be exploited as an actor in the maintenance of a transient cellular state in EMT progress towards metastatic development of breast tumors.

## Introduction

Triple-Negative Breast Cancer (TNBC) is clinically defined by the lack of expression of the estrogen (ER) and progesterone (PgR) receptor genes, and by the absence of amplification of human epidermal growth factor receptor-2 (HER2). Treatment of TNBC has been challenging due to its heterogeneity at the molecular level and the absence of well-defined molecular targets [1], [2]. Despite a frequent complete response to neoadjuvant chemotherapy, TNBC patients also have a higher rate of long term recurrence and worse prognosis than ER-positive BC patients. Distinguishing chemoresistant TNBC patients at risk to relapse from those with a relatively favorable prognosis, would help to identify clinically relevant subgroups that could benefit from alternative treatments.

Advances in gene expression profiling have permitted characterization of different intrinsic molecular subtypes present in TNBC [3]. One of these, the claudin-low breast cancer subtype [4], is characterized by mesenchymal features, low expression of cell-cell junction proteins (i.e., E-cadherin), and intense immune infiltrates. Furthermore, claudin-low tumors have unique biological properties linked to mammary stem cells [5] and Epithelial-Mesenchymal Transition (EMT) features [6].

Gene expression during EMT is dependent on specific transcription factors that interact with enhancer or promoter elements, the accessibility of their binding sites which is regulated by epigenetic reprogramming [7], [8]. Hence, chromatin reorganization could contribute to the regulation of epithelial plasticity [9]–[12]. To date however, the presence of histone variants has not been investigated with respect to the phenomenon of EMT. Gene expression accompanying EMT is also regulated at the post-transcriptional level via alternative splicing of RNA [13]–[15].

The histone variant macroH2A1 is a vertebrate-specific member of the H2A family and is unusual due to the presence of a C-terminal macro domain [16]. Two isoforms, macroH2A1.1 and macroH2A1.2 are produced by alternative splicing of the *H2AFY* gene. Both isoforms have been associated with silencing and transcriptional repression [17]–[19]. Regulation of macroH2A1 expression seems to be linked to self-renewal and commitment of ES cells, representing a barrier to reprogramming pluripotency [20]–[22]. In melanoma, loss of macroH2A1 promoted progression of metastasis [23]. Moreover, high levels of macroH2A1.1 are associated with slowly proliferating cancers, whereas highly proliferating tumors have markedly decreased macroH2A1.1 levels. Conversely, macroH2A1.2 expression is independent of proliferation in all tumours [24]–[26]. Notably, expression of macroH2A1.1 has been identified as a novel biomarker in lung and colon cancer models [25], [26].

In this study, we demonstrate that selective splicing of the *H2AFY* gene is correlated with EMT features linked to Claudin-low breast cancers. We propose that macroH2A1.1 expression levels could participate in the epigenetic program linked to poor clinical outcome of this molecular breast cancer subtype, and more generally in the EMT process.

## Materials and Methods

### Cell culture

MCF-7 and MDA-MB231 were obtained from ATCC. ZR-75, MDA-MB436 and Hs578T, were a gift from G. Freiss (Montpellier, France), originally purchased from ATCC [27]. MDA-MB231, MDA-MB436 and Hs578T cells were maintained in DMEM high glucose with glutamax. MCF-7 cells were maintained in DMEM/F12 with Glutamax. ZR-75 cells were maintained in RPMI-1640 supplemented with 10 mM Hepes. All these media were supplemented with 10% heat-inactivated fetal bovine serum and 1 mM sodium pyruvate.

### Protein quantification

Antibodies against macroH2A1 (07-219; Upstate), macroH2A1.1 and macroH2A1.2 (gift by A. Ladurner), ERα (sc-543; Santa Cruz), GAPDH (MAB374; Millipore), H3 (ab1791; Abcam) were used for immunoblotting. To discriminate between the two splicing isoforms of macroH2A1, macroH2A1.1 and macroH2A1.2, total cell extracts were separated on low cross-linking (12% acrylamide,1∶125 bisacrylamide) SDS-polyacrylamide gels and blotted with antibodies specific to one of the two isoforms (Fig.S1) specifically. Proteins were quantified using the Image Gauge software. Expression levels of each isoform and total macroH2A1 were normalized to GAPDH. ZR-75 expression was used as sample reference.

### RNA extraction, reverse transcription and Quantitative PCR analysis

Total RNA was extracted using an RNeasy mini kit (Qiagen). First strand cDNA was generated using the ThermoScript RT-PCR system (Invitrogen) and used as the template for quantitative PCR (qPCR) using the platinium SYBR Green qPCR SuperMix (Invitrogen) according to the manufacturer's instructions. Gene- or splice variant-specific primers are shown in Fig.S2A. Relative levels of RNA were determined using the threshold cycle (Δ*C_T_*) method [28]. Expression levels were normalized to the ribosomal RPLP0 gene. ZR-75 expression was used as sample calibrator.

### Determination of the macroH2A1.1/macroH2A1 mRNA ratio

A summary of probe set IDs used is reported in Fig.S2. Among the probe set ID from HG-U133A, three detect the expression of *H2AFY* gene: 214501_s_at and 207168 _s_at probe set ID which are common to the two isoforms and 214500_at which recognized specifically the sequence of the exon 6a of macroH2A1.1 and 10 nucleotides in exons 5 and 7 common to macroH2A1.1 and macroH2A1.2 isoforms (Fig.S2). We extracted the corresponding log2 RMA values from the different GEO datasets studied and determined relative expression of macroH2A1 (mean value of 214501_s_at and 207168_s_at), macroH2A1.1 (214500_at) and calculated the macroH2A1.1/macroH2A1 mRNA ratio by the following formula: 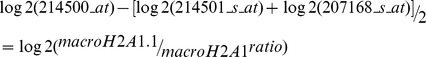



Among the probe set ID from Illumina Human-6 v1 expression bead chip, two of them detect the expression of *H2AFY* gene: 6620403 probe set ID which is common to the two isoforms and 6620403 which recognized specifically the sequence of the exon 6a of macroH2A1.1. Among the probe set ID from Illumina HumanHT-12 v4.0 expression bead chip, three detect the expression of *H2AFY* gene: ILMN_2373495 and ILMN_1746171 probe set ID which are common to the two isoforms and ILMN_1674034 which recognized specifically the sequence of the exon 6a of macroH2A1.1. We applied the same formula as above with corresponding log2 RMA values.

### Analysis of correlation of macroH2A1.1/macroH2A1 mRNA ratio and breast cancer cell lines markers

For each GEO dataset analyzed, we conserved the intrinsic molecular subtype of breast cancer cell lines attributed in the original study or in absence of we attributed the molecular intrinsic subtype as defined in Table S1. Then we classified the different breast cancer cell lines into two groups, luminal/basal, or claudin-low/non claudin-low and compared the distribution of macroH2A1, macroH2A1.1 expression levels or macroH2A1.1/macroH2A1 mRNA ratio values (Table S2). The reported *p*-values are the results of a two-tailed Mann-Whitney test.

### Survival analyses

A summary of affymetrix microarray datasets used in this study, including the number of patients included in each stage of the analysis, is given in Table 1.

**Table 1:** Summary of affymetrix microarray datasets used in this study.

		% of samples (complete datasets)						No of samples								
Dataset	Data Source	Tumor size ≤ 2cm	LNN	G3	Systemic Treatment			Total	TNBC	DMFS	RFS		Used in			*Ref*
					No	Yes										
						endocrine treatment	chemotherapy						All samples	Untreated	Treated	
San Francisco	E-TABM-158	35	52	45	17	13	70	118	23	x			23	7	16	[46]
Mainz	GSE11121	33	100	71	100	0	0	200	21	x			21	21		[47]
Veridex-Tam	GSE12093		100		0	100	0	136	1	x			1	1		[48]
Rotterdam-EMC204	GSE12276	22		71	63	25	13	204	56		x		56	42	6	[49]
Stockholm	GSE1456			74	45	55	0	159	25		x		25	22		[50]
Rotterdam-EMC344	GSE2034, GSE5327	35	100	72	100	0	0	344	82	x			82	82		[51] [52]
New York	GSE2603	11	49		0	4	96	99	35	x			29	1	26	[53]
Oxford-untreated	GSE2990		100		100	0	0	61	13		x		11	11		[54]
Hamburg-2	GSE31519	0	71	40	0	100	0	77	7	x			5	5		[55]
Hamburg-1	GSE31519	33	80	86	0	0	100	77	15		x		15		15	[56]
Franckfurt-2	GSE31519	0	39	56				67	19		x		18	14		[57]
Franckfurt-3	GSE31519	50	50	50	0	100	0	52	2		x		2	2		[58]
Frankfurt	GSE31519	42	83	83	0	0	100	119	24		x		24		24	[59]
Uppsala	GSE3494	41	68	59	86	14	0	251	27		x		27	21		[60]
London	GSE6532	0	0	100	0	100	0	87	2		x		2	2		[61]
TransBIG	GSE7390	18	100	75	100	0	0	198	40		x		40	40		[62]
London-2	GSE9195	100	100	100	0	100	0	77	2		x		2	2		[63]
***TOTAL:***													***383***	***273***	***87***	

*Adapted from Rody A et al.,* [*29*] *.*

Follow-up data was available for 383 of the 579 TNBC samples from the GSE31519 dataset. All survival intervals were measured from the time of surgery to the distinct survival endpoint used in the individual datasets. In the conduct of the presented analysis event free survival (EFS) was calculated as preferentially corresponding to the RFS endpoint, but measured with respect to the DMFS endpoint if RFS was not available. Rody A. et al., [29] have previously shown that the effect of using these different endpoints was rather small in the overall dataset. Follow up data for those women in whom the envisaged end point was not reached were censored as of the last follow-up date or at 120 months. Subjects with missing values were excluded from the analyses. For the analyses of untreated and adjuvant therapy treatment groups, we applied the Kolmogorov-Smirnov test to compare the cumulative distribution of the two data sets.

To determine the cutoff value of macroH2A1.1/macroH2A1 mRNA ratio, a receiver-operating characteristics (ROC) analysis was performed. We constructed Kaplan-Meier curves and used the log-rank test to determine the univariate significance of the variables. The predictive potential of macroH2A1.1/macroH2A1 mRNA ratio is assessed by its positive and negative predictive values (PPV and NPV) (Table S3). A Cox proportional-hazards model was used to examine the effects of multiple covariates on survival (Table S3). All *P*-values are two-sided and 0.05 was considered as a significant result.

All statistical analyses were performed using the XLSTAT version 2013.1.

### Accession codes

Summary of public databases used: Gene Expression Omnibus (NCBI, Bethesda, MD, USA): GSE16795, GSE9691, GSE24202, GSE31519; ArrayExpress (EBI, Hinxton, UK): E-TABM-157, E-MTAB-183, E-MTAB-827, E-MTAB-884.

## Results

### Expression of MacroH2A1 splice variants in breast cancer cell lines

**Figure 1:**
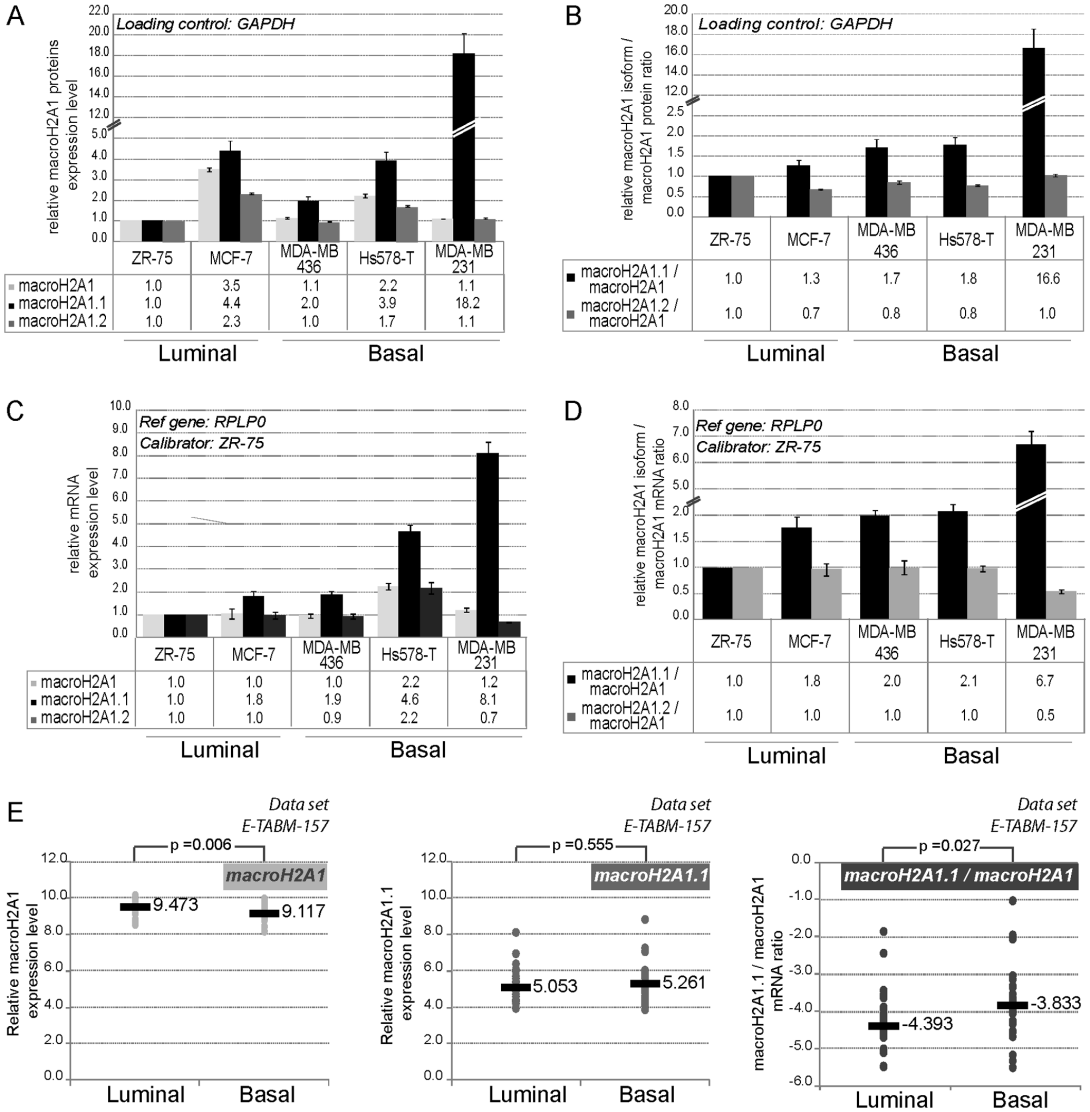
Expression levels of macroH2A1 splice variants in breast cancer cell lines. **A-B-** MacroH2A1.1, macroH2A1.2, and total macroH2A1 protein expression levels in five breast cancer cell lines. Quantification of each macroH2A1 splice variants, global macroH2A1 (A) and macroH2A1.1/macroH2A1, macroH2A1.2/macroH2A1 ratios (B) for each cell line normalized to GADPH is shown relative to the ZR-75 cell line. **C-D-** qPCR analysis of mRNA expression levels of macroH2A1, macroH2A1.1, macroH2A1.2 and macroH2A1 splice variants/macroH2A1 mRNA ratio. Each quantification was performed at minimum in biological triplicate. Expression levels were normalized to expression of RPLP0, and referred to the cell line ZR-75 as a sample calibrator. **E-** Analysis of expression data of macroH2A1 variants in 51 breast cancer cell lines on the basis of U133A array hybridization [30]. Log2 macroH2A1, MacroH2A1.1 and macroH2A1.1/macroH2A1 values are determined and classified according to luminal or basal molecular subtype as defined in [30]. Data of DU4475, HCC1008 and HCC1599 are included in the analysis with the molecular subtype assigned in the synthesis part of Table S1. The median of each subgroup is shown (grey bar). The reported *p*-values are the results of a two-tailed Mann-Whitney test.

We quantified protein expression levels of macroH2A1, macroH2A1.1, macroH2A1.2 *per se* and each of the macroH2A1 isoforms relative to the total macroH2A1 protein pool in BC cell lines. MacroH2A1 isoforms or macroH2A1 expression levels did not differ significantly between cell lines representative of the ER positive luminal subtype, ZR-75 and MCF-7 cells, and cell lines of the ER negative basal subtype, MDA-MB436, Hs578T, and MDA-MB231 (Fig.1A). In contrast, expression levels of macroH2A1.1 protein relative to total macroH2A1 protein pool were greater in cells of the basal subtype compared to the luminal one (Fig.1B). Unlike macroH2A1.2, which showed no significant variation in expression levels, the increase in macroH2A1.1 was greatest in MDA-MB231 cells.

Using splice variant-specific primers (Fig.S2), we also determined that macroH2A1.1 transcription was greater in the basal compared to the luminal subgroup of BC cell lines, while mRNA expression levels of macroH2A1.2 or total macroH2A1 mRNA did not differ significantly (Fig.1C). As at the protein level, this differential expression was consolidated by analysis of the proportional expression of macroH2A1.1 relative to total macroH2A1 (Fig.1D).

To test whether this observation could be extended to a larger panel of BC cell lines, we analyzed macroH2A1 expression in 51 BC cell lines from data published by Neve *et al.,* [30] (Fig.1E). Total macroH2A1 expression levels were reduced in the basal BC cell lines (*p = 0.006*). Therefore, even if macroH2A1.1 expression levels *per se* did not vary (*p = 0.555*), the relative proportion of macroH2A1.1 to global macroH2A1 was significantly higher in the basal subtype (*p = 0.027*).

**Figure 2:**
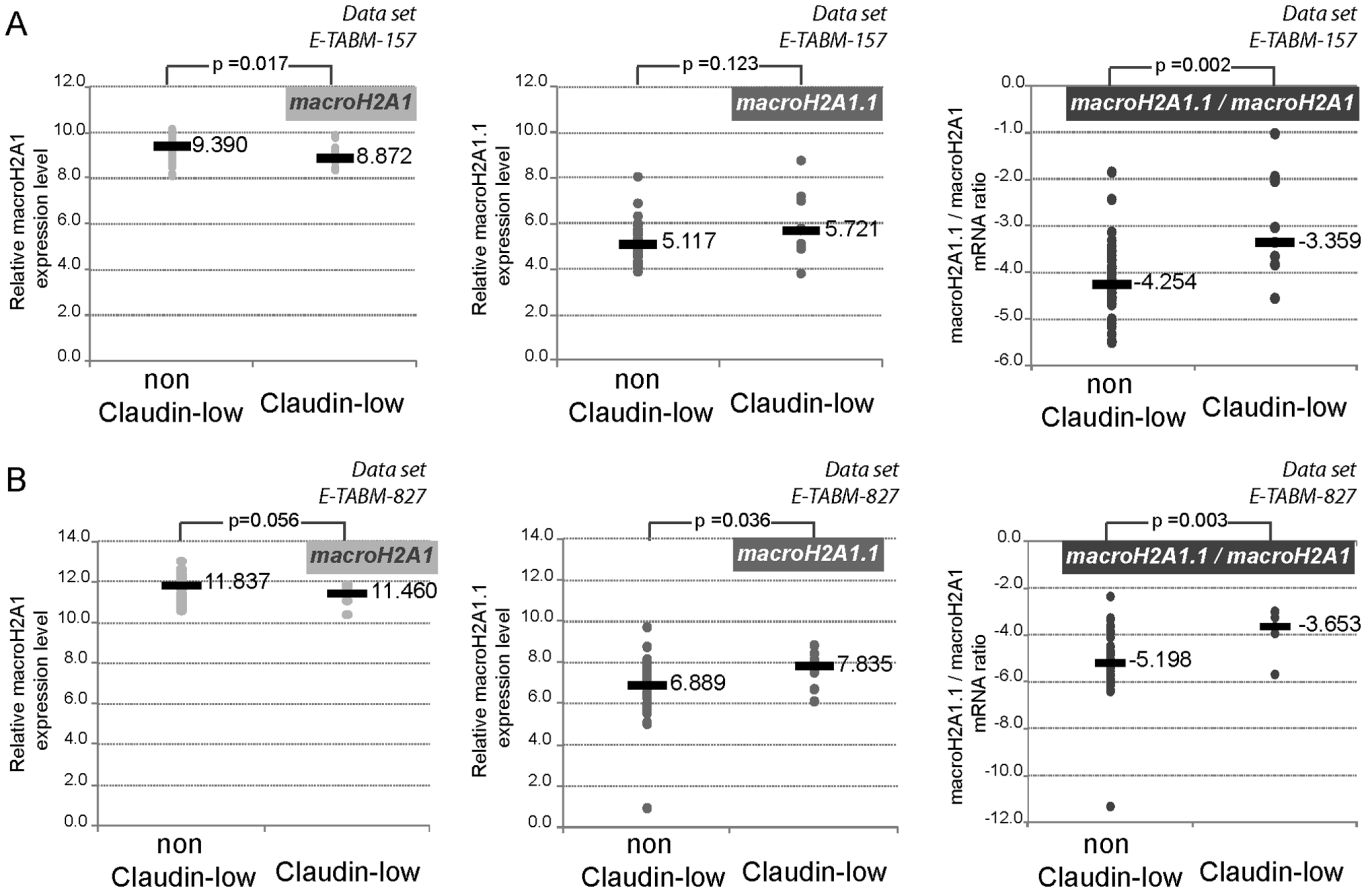
High macroH2A1.1 expression level in breast cancer cell lines characterizes Claudin-low molecular subtype. In two independent analyses, macroH2A1, macroH2A1.1 and macroH2A1.1/macroH2A1 mRNA ratios were determined for each cell line and classified according to claudin-low or non claudin-low molecular subtype assigned in the synthesis part of Table S1. The median of each subgroup are specified. In E-TABM-827 analysis, GI-101, HB4A, PMC42 and VP229 cell lines were omitted as the subgroup Basal A or B was not specified; HCC1509, MT3 and VP267 cell lines are omitted as their subtype were not assigned. The reported *p-*values are the result of a two-tailed Mann-Whitney test.

Because the three basal BC cell lines tested (Fig.1A–D) belonged to the claudin-low subtype, we subdivided the cell lines from Neve et al., study into two groups: claudin-low and non claudin-low BC cell lines. Cell line subtypes were attributed as in Prat et al., [31] (Table S1). Then we compared the relative expression levels of macroH2A1 mRNAs. Increased macroH2A1.1 expression levels appeared typical of claudin-low subtype BC cell lines (Fig.2A). This correlation was significant for the macroH2A1.1/macroH2A1 mRNA ratio (compare *p-values* Fig.2A center and right panels) and was further confirmed by our analysis of several independent studies that differed in the nature of the cell lines (Fig.S3) and the array platform used (Fig.2B).

**Figure 3:**
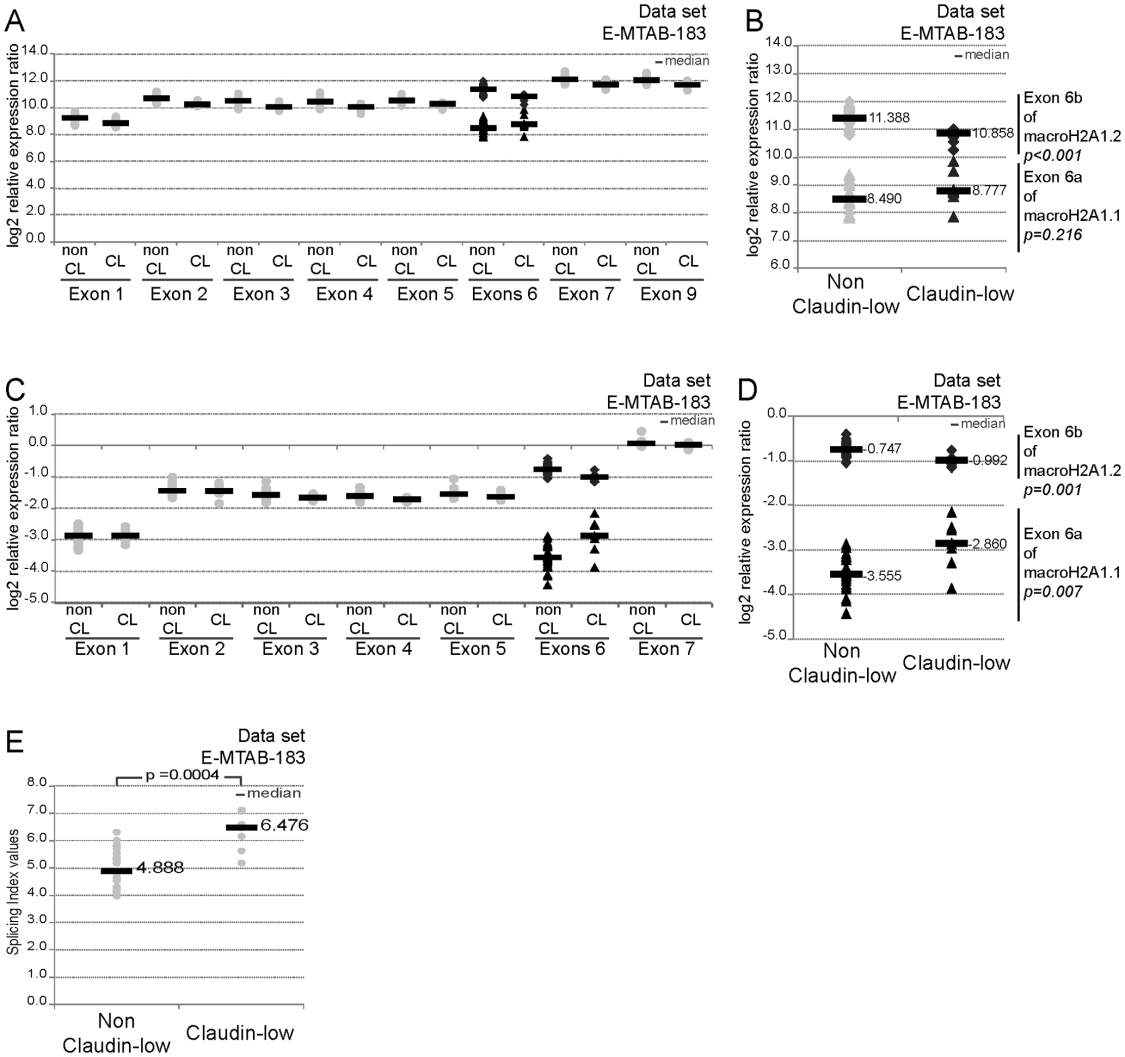
Analysis of expression data of macroH2A1 variants in breast cancer cell lines on the basis of Affymetrix Human Junction technology (E-MTAB-183[11]). A- Log2 expression values of exons of H2AFY gene are represented by molecular subtype non claudin-low (non CL) and claudin-low (CL). The analysis for exons 6a/b are highlight in B. C- Log2 expression values of exons of H2AFY gene normalized to the one of exon 9 are represented by molecular subtype non claudin-low (non CL) and claudin-low (CL). The analysis for exons 6a/b are highlight in D. E- Splicing index values for exon 6a included in macroH2A1.1 splice variant are represented by molecular subtype. The median of each subgroup is shown (grey bar). The reported *p*-values are the results of a two-tailed Mann-Whitney test.

Finally, the study of Lapuk et al. [32] allowed us to assess alternative splicing in 31 BC cell lines. We first analyzed relative expression levels of each exon of *H2AFY* gene except that of exon 8 (data unavailable). Globally each exon was less expressed in the claudin-low than in the non-claudin low BC cell lines (Fig.3A). One exception appeared to be the exon 6a of macroH2A1.1 which was expressed more strongly in Claudin-low BC cell lines, but the difference was not statistically significant (Fig.3B). We normalized the level of expression of each exon (log2 RMA values) relative to that of exon 9, the most expressed exon of the *H2AFY* gene. As shown in Fig.3C, expression levels of most of the exons of the *H2AFY* gene in claudin-low subtype cells decreased; and this was also true for the macroH2A1.2 specific exon 6b (*p = 0.001*) (Fig.3D). In contrast, expression levels of macroH2A1.1 specific exon 6a increased in the claudin-low compared to the non claudin-low subtype (*p = 0.007*). Moreover, determination of the splicing index showed that only exon 6a varied in all cell lines tested (Fig.3E).

We conclude that the expression of macroH2A1.1 relative to total macroH2A1 expression (macroH2A1.1/macroH2A1 mRNA ratio defined in materials and methods), not macroH2A1.1 expression *per se*, is correlated specifically with the claudin-low molecular subtype.

### MacroH2A1.1 variant expression correlates with epithelial-mesenchymal transition

**Figure 4:**
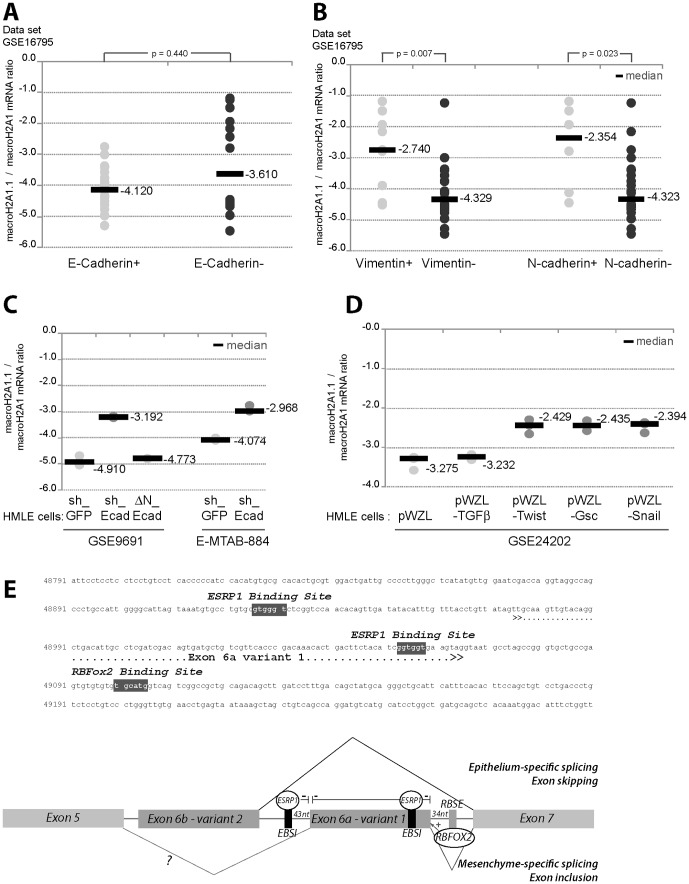
Overrepresentation of macroH2A1.1 correlated with mesenchymal features and induction of EMT features in HMLE cells is accompanied by an increase in macroH2A1.1/macroH2A1 mRNA ratio. A–B Analysis of GSE16795 data set [33]. MacroH2A1.1/macroH2A1 mRNA ratio are determined for each cell line and classified depending of the level of expression of E-cadherin (A); of the level of expression of Vimentin or N-cadherin (B). The median of macroH2A1.1/macroH2A1 values of each subgroup are specified. The reported *p-*values are the result of a two-tailed Mann-Whitney test. C- macroH2A1.1/macroH2A1 mRNA ratios were determined in immortalized human mammary epithelial cells with inhibiting E-cadherin function either shRNA-mediated (GSE9691 [34] and E-MTAB-884 [45]) or by expression of a truncated form of E-cadherin (ΔN-Ecad) (GSE9691 [34]) and compared. D- MacroH2A1.1/macroH2A1 mRNA ratios were determined for different breast cancer stem cell-like lines which overexpressed one EMT inducer, *i.e.* TGFβ, Twist, Gsc or Snail, and compared (GSE24202 [6]). The median of each subgroup is shown (grey bar). The reported *p*-values are the results of a two-tailed Mann-Whitney test. E- Upper panel- genomic sequence of H2AFY gene encompassing exon 6a. Potential ESRP1 and RBFox2 binding sites are represented with grey background and white letters. Bottom panel- Hypothetical schema for alternative splicing of exon 6a included in the macroH2A1.1 splice variant. Two cellular backgrounds are represented, epithelial with exon skipping of exon 6a related to the inhibitory binding of ESRP1 to EBSI, and mesenchymal with exon inclusion of exon 6a potentiated by binding of RBFox2 to RBSE.

We classified the macroH2A1.1/macroH2A1 mRNA ratios of a set of 38 BC cell lines relative to the E-cadherin expression data determined by Hollestelle *et al*., [33]. In E-cadherin^negative^ cell lines this ratio was generally greater and more diverse, but the difference between E-cadherin^positive^ and E-cadherin^negative^ cells was not statistically significant (Fig.4A). Different mechanisms for inactivating E-cadherin have been identified in human cancers: inherited and somatic mutations, increased promoter methylation, and induction of transcriptional repressors of the Twist, Snail and Zeb family members [6]. The latter induce EMT in parallel with induction of mesenchymal markers such as N-cadherin and/or vimentin. Interestingly, most of the cell lines exhibiting high macroH2A1.1/macroH2A1 mRNA ratios, expressed N-cadherin and vimentin (Fig.4B).

In order to determine whether enrichment of macroH2A1.1 could be related to the EMT process, we analyzed expression levels of this variant in different cellular models of EMT. Comparison of macroH2A1.1/macroH2A1 mRNA ratios in HMLE_shGFP and HMLE_shEcad, revealed that reduction of E-cadherin expression levels was accompanied by an increase in the macroH2A1.1 mRNA ratio in two independent data sets (Fig.4C) [34], [35]. This increase was clearly associated with induction of EMT due to dysfunction in intracellular signaling caused by reduced E-cadherin levels. Indeed, expression of a truncated form of E-cadherin (ΔN-Ecad) lacking the extracellular domain of the wild-type protein normally responsible for E-cadherin cell-cell adhesion was not correlated with an increase in macroH2A1.1mRNA ratios (Fig.4C). Accordingly, overexpression of inducers of EMT, Twist1, Goosecoid or Snail in the HMLE cell line was accompanied by an increase in macroH2A1.1 mRNA (Fig.4D). In contrast, macroH2A1.1 mRNA ratios were not up-regulated by overexpression of TGF-β. This is in agreement with previous observations showing that induction of an EMT by Snail or Twist does not depend on TGF-β autocrine signaling [6]. Moreover, TGF-β signaling is not sufficient for an EMT conversion in primary normal, immortalized, and neoplastic HMECs [36], and is thus insufficient to induce an increase in macroH2A1.1/macroH2A1 mRNA ratio.

Extensive changes in alternative splicing play a role in shaping cellular behavior patterns that characterize EMT. Interestingly, the macroH2A1.2 specific exon was shown to be an Epithelial Splicing Regulatory Protein (ESRP)-regulated cassette [4]. Analysis of the genomic context of the exon specifically included in macroH2A1.1 identified potential binding sites for the EMT-associated splicing factors, ESRP1 and RBFOX2 (Fig.4E). ESRP binding sites located at the 5′ end and within the regulated exon seem to be ESRP Binding Splicing Inhibitors (EBSI), and a RBFOX2 binding site found downstream of the alternatively spliced exon seems to be an RBFOX2 Binding Splicing Enhancer (RBSE). Exon 6a skipping could thus result from the interaction of ESRP1 with EBSI in an epithelial context. In a mesenchymal context, exon 6a would be preferentially included by enhanced binding of RBFOX2 (Fig.4E).

### Prognostic significance of macroH2A1.1 mRNA ratio in TNBC

To assess the potential prognostic value of macroH2A1.1 mRNA ratio in breast cancer, we analyzed the event-free survival of patients as a function of macroH2A1.1 mRNA ratio reported for the GSE31519 dataset, which provides access to a large cohort of TNBCs (Table 1).

**Figure 5:**
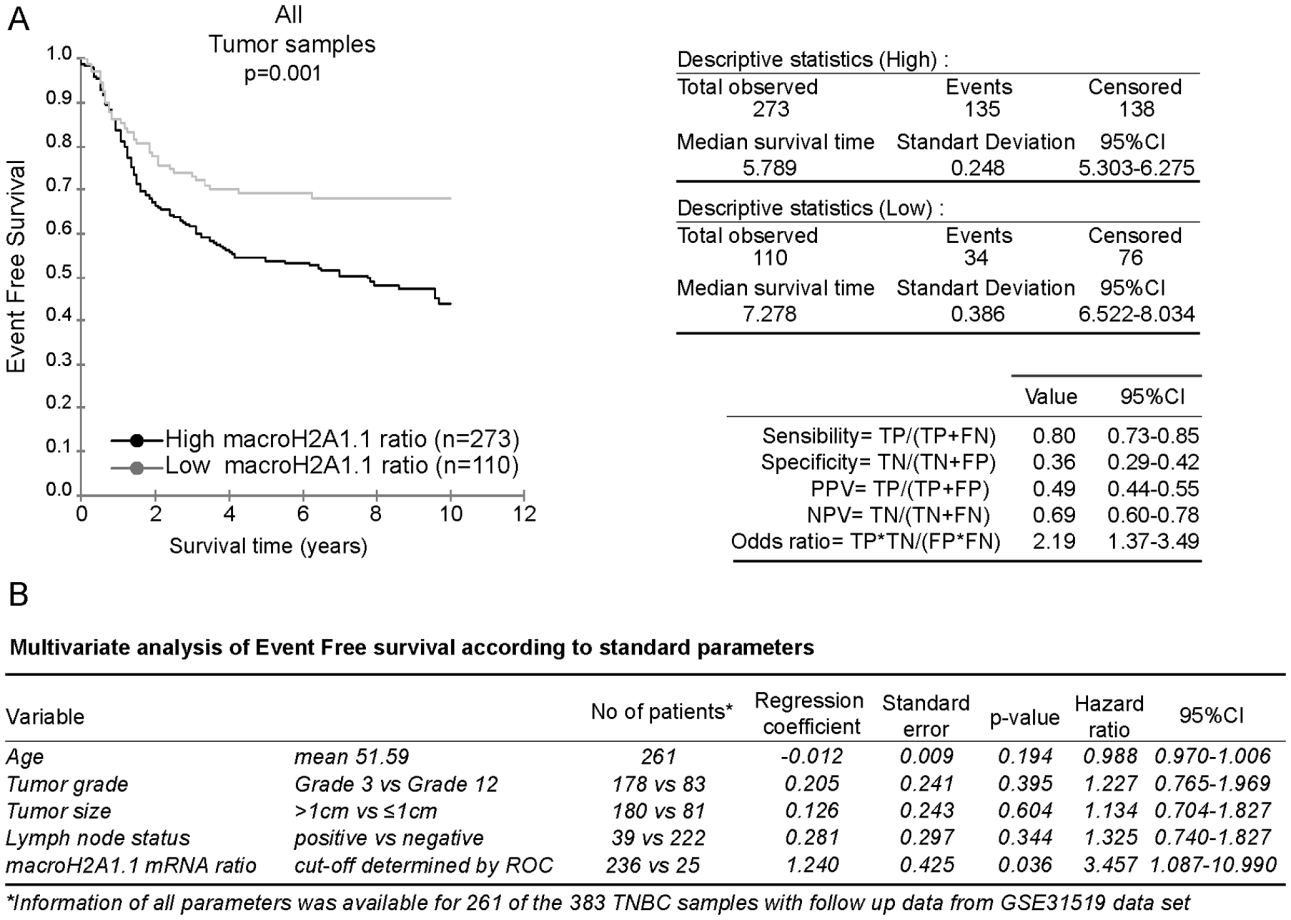
Kaplan Meier analysis according to the macroH2A1.1 mRNA ratio. **A-** The 383 TNBC samples from the GSE31519 cohort were stratified according to the macroH2A1.1/macroH2A1 mRNA ratio. Kaplan Meier analysis of event free survival of 383 samples with follow up information is shown. Positive and negative predictive values (PPV and NPV) of macroH2A1.1/macroH2A1 mRNA ratio in the cohort are specified. **B-** Multivariate Cox proportional hazards models of disease-free survival.

We plotted Kaplan-Meier survival curves based on macroH2A1.1 mRNA ratios segmented into two groups, high and low macroH2A1.1 mRNA ratios. The Receiver Operating Characteristic (ROC) analysis was used to find the optimal cut-off level. We used the log-rank test to determine the univariate significance of the variables. Poor prognosis TNBCs had high macroH2A1.1 mRNA ratios (p = 0.001) (Fig.5A). The positive predictive value (PPV) and negative predictive value (NPV) were 49% and 69%, respectively. In multivariate Cox regression analysis, including age, histological grade, tumor size and lymph node status, the macroH2A1.1/macroH2A1 mRNA ratio showed a trend as an independent predictor (HR 3.457, 95%CI 1.087 to 10.990, *p = 0.036*) (Fig.5B).

**Figure 6:**
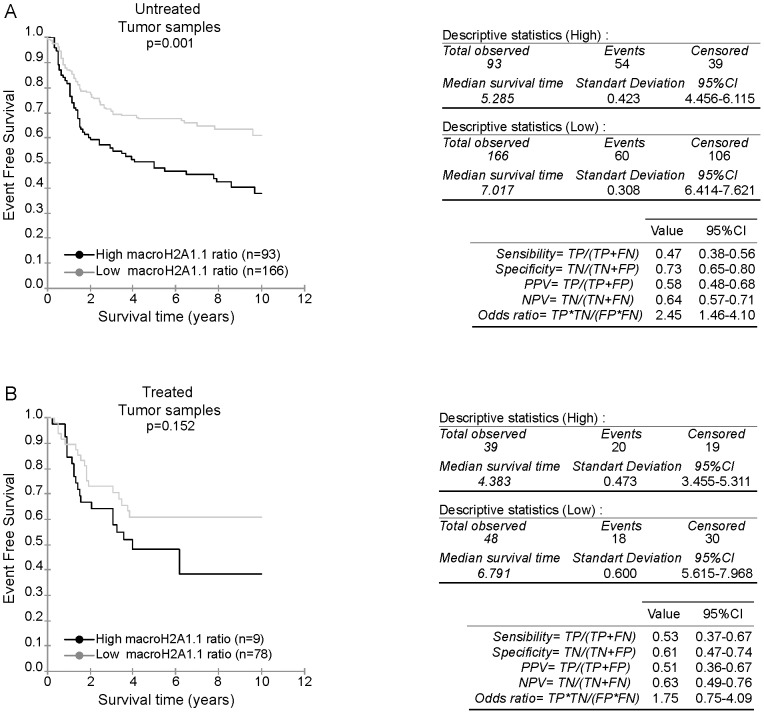
Kaplan Meier analysis according to the macroH2A1.1 mRNA ratio in untreated and treated sub-cohort. **A-** The 259 TNBC untreated samples from the GSE31519 cohort were stratified according to the macroH2A1.1/macroH2A1 mRNA ratio. Kaplan Meier analysis of event free survival of 259 samples with follow up information is shown. PPV and NPV of macroH2A1.1/macroH2A1 mRNA ratio in the untreated cohort are specified. **B-** The 87 TNBC adjuvant chemotherapy treated samples from the GSE31519 cohort were stratified according to the highest macroH2A1.1/macroH2A1 mRNA ratio. Kaplan Meier analysis of event free survival of 87 samples with follow up information is shown. PPV and NPV of macroH2A1.1/macroH2A1 mRNA ratio in the treated cohort are specified.

Among the clinico-pathological characteristics of TNBC patients included in the GSE31519 dataset, the use of systemic treatment was specified. Hence, we sub-divided the cohort into one untreated sub-cohort, and a second treated sub-cohort which regroups all adjuvant treated patients. Analysis of the macroH2A1.1/macroH2A1 mRNA values in the untreated sub-cohort revealed a distribution comparable to the values of the total cohort (*p = 0.235*) (Fig.S4). MacroH2A1.1/macroH2A1 mRNA values of the group treated with an adjuvant therapy differed from those of the total cohort, with values shifted to higher values of the intervals (*p = 0.008*) (Fig.S4). Kaplan-Meier survival curves were plotted according to macroH2A1.1 mRNA ratios after segmentation into high and low groups as above for the two sub-cohorts. We observed that in contrast to treated tumors, high macroH2A1.1 mRNA ratios still correlated with reduced survival curves for untreated tumor (p = 0.152 vs. p = 0.001) (Fig.6).

## Discussion

We provide evidence that overexpression of macroH2A1.1 correlates with major mesenchymal markers of the claudin-low breast cancer subtype. Notably, the increase in macroH2A1.1 seems to be a residual track of an EMT process, correlated with poor prognosis in TNBCs.

Claudin-low tumors are typically TNBCs with poor long-term prognosis, despite reduced expression of genes related to cell proliferation. Nevertheless, unlike prognostic signatures that rely heavily on proliferation-related genes, macroH2A1.1 preferentially associated with non-proliferative phenomena. It would belong to a new prognostic marker class independent of proliferative status, similar to factors related to the immune system response [37].

Interestingly, it was shown that upon entering EMT HMECs develop a stable, low proliferative mesenchymal phenotype. MacroH2A1 was identified as an epigenetic barrier which participates in the maintainance of cell identity and antagonizes induction of cell reprogramming to naive pluripotency [38], [39]. Thus, macroH2A1.1 could be involved in the maintenance of a mesenchymal state, partial or complete, by establishing an epigenetic barrier against further de/differentiation.

The difficulty of identifying EMT-transitioning cells *in vivo* creates skepticism regarding the pathological relevance of EMT. One explanation for this is that cancer cells only undergo a transient EMT, reverting back to the epithelial state by a mesenchymal-epithelial transition (MET), making it difficult to isolate cells with true EMT markers. Studies in experimental mouse models have shown that a complete EMT-MET cascade is important for tumor metastasis [40], [41]. If the EMT process is so transient and, in parallel, so important for the development of metastatic tumors, why do only claudin-low and, to a lesser extent, metaplastic intrinsic molecular subtypes of BC present molecular features of EMT? One explanation could be that in claudin-low tumors the EMT-MET turnover is trapped in an intermediate mesenchymal state, in which EMT markers are present. We speculate that macroH2A1.1 stabilizes chromatin organizations characteristic of transcriptional programs linked to paused cell cycle progression. Hence, macroH2A1.1 expression could divert EMT-MET processes, stop progression and trap cells in such an intermediate state.

High macroH2A1.1 mRNA ratios in the slow cycling claudin-low molecular subtype [31] are correlated with earlier observations that macroH2A1.1 expression may be restricted to non-proliferative tissues [42], and that loss of its expression in lung and colon cancer was related to enhanced cell proliferation of cancer cells [24]–[26]. In the 67NR mouse model which formed primary carcinomas when implanted into mouse mammary fat pads, Dardenne et al., identified a high macroH2A1.1/macroH2A1 ratio. Inversely, in the 4T1 mouse model, reduced macroH2A1.1 expression was correlated with macroscopic metastatic capacity in the lung [43]. Our results point to high macroH2A1.1/macroH2A1 ratios as markers of engaged but paused intermediate cellular stages of the EMT. Because the metastasic power of a tumor clearly depends on a complete EMT-MET process, it is tempting to propose a model in which macroH2A1.1 is linked to the EMT process and macroH2A1.2 linked to the MET process.

TNBC is generally associated with a poor outcome, which is essentially not predicted by assessment of standard clinico-pathological variables, such as lymph node status or tumour size at initial presentation. The lack of identified molecular targets in the majority of TNBCs implies that chemotherapy remains the treatment of choice for patients with TNBCs. Here we show that, regardless of the reason that led to an absence of adjuvant therapy for patients involved in the GSE31519 study, those with a high macroH2A1.1/macroH2A1 mRNA ratio have a worse prognosis than those with a low one. Even if this observation clearly needs to be confirmed with a larger cohort, it is tempting to propose that assessing macroH2A1.1 expression levels will allow the identification of TNBC patients who, despite favorable clinic-pathological variables such as lymph node status or tumour size at initial presentation, will have a worse prognosis and may benefit from treatment. Interestingly, EMT and cell dissemination, although long associated with advanced stage of tumor progression, can be found at pre-neoplastic developmental stages of tumors [44]. Identifying early EMT process in primary tumors could then allow detection of tumors progressing towards metastasis. As expression of macroH2A1.1 seems to be correlated with EMT and unfavorable behavior in untreated TNBC patients, it is tempting to suggest macroH2A1.1 expression levels as an early biomarker of tumor genesis.

No difference in survival of patients who underwent adjuvant treatment was seen with respect to the macroH2A1.1/macroH2A1 mRNA ratio. But, as the ratios of this sub-cohort are already globally higher than in untreated tumors (Fig.S4), one could speculate that clinico-pathological parameters that initially led to treatment may already correlate with higher macroH2A1.1/macroH2A1 mRNA ratio. It will be interesting to analyse this more in depth in a larger cohort.

In conclusion, it will be tempting to test if the correlation between macroH2A1.1 expression levels and EMT markers or poor prognosis in a TNBC cohort could be linked to a role for macroH2A1.1 in the maintenance of a transient cellular state in the early EMT process towards metastatic development of breast tumors.

## Supporting Information

Figure S1 **A-** Characterization of α-macroH2A1 antibodies and cell lines. The specificity of α-macroH2A1 antibodies was verified using SDS-polyacrylamide gels low cross-linking (12.5% acrylamide, 1∶125 bisacrylamide) to separate the two splice variants macroH2A1.1 and macroH2A1.2. Total extracts of breast cancer cell lines were first resolved in SDS-polyacrylamide gels (standard (left) or low cross-linking (right panel)) then immunoblotted with α-macroH2A1 and α-ERα antibodies. Left panel: ERα, macroH2A1 and H3 specific antibodies. Right: top panel; macroH2A1 specific; middle panel: macroH2A1.1 specific; bottom panel: macroH2A1.2 specific antibody. **B-** MacroH2A1.1, macroH2A1.2, and total macroH2A1 protein expression levels in five breast cancer cell lines. Total protein extracts were immunoblotted with α-macroH2A1 (bottom panel), α-macroH2A1.1 (top panel) or α-macroH2A1.2 antibodies (middle panel).
(TIF)

Figure S2 **Primers, qPCR parameters and probe set IDs summary.** **A-** Sequences, genomic location and size of amplicons generated by primers used in qPCR reactions are summarized. Parameters of the standard curve are reported for each pairs of primers in each cell lines used. For a given amplicon, efficiencies in the different cell lines are reported and compared each other (STDEV(Es)). **B-** Characterization of probe set ID of Affymetrix U133A, Illumina Human-6 v1 expression beadchip, IlluminaHuman HT-12 v3.0 expression beadchip arrays corresponding to macroH2A1 variants. For each probe, nucleotide reference sequences and macroH2A1 isoforms recognized are reported, as nucleotide and genomic localization of the sets of oligonucleotides presents at the probe ID. **C-** Clustal W multiple alignment of macroH2A1 variants sequences represented at the Probe Set ID 214500_at from U133A array. The sequences recognized by the probe are highlighted.
(TIF)

Figure S3 **High macroH2A1.1 expression level in breast cancer cell lines characterizes Claudin-low molecular subtype.** MacroH2A1.1/macroH2A1 mRNA ratios were determined for each cell line and classified according to molecular subtype assigned in the synthesis part of Table S1. In GSE16795 analysis [33], data from H3396 cell line are omitted as its subtype was not assigned. The median of macroH2A1.1/macroH2A1 values of each subgroup are specified. The reported *p-*values are the result of a two-tailed Mann-Whitney test.
(TIF)

Figure S4 **Analysis of the distribution of macroH2A1.1/macroH2A1 mRNA values in the different groups of patients studied.**
(TIF)

Table S1 **Summary of molecular subtype of breast cancer cell lines used in this studies.**
(PDF)

Table S2 **Selected GEO dataset for cell lines analysis.**
(XLSX)

Table S3 **GSE31519 analysis.**
(XLSX)
