# Supplementary figures and images for: Increased macroH2A1.1 Expression Correlates with Poor Survival of Triple-Negative Breast Cancer Patients
Source: PLoS One. 2014 Jun 9;9(6):e98930. doi: 10.1371/journal.pone.0098930 (PMC4049614; doi:10.1371/journal.pone.0098930)

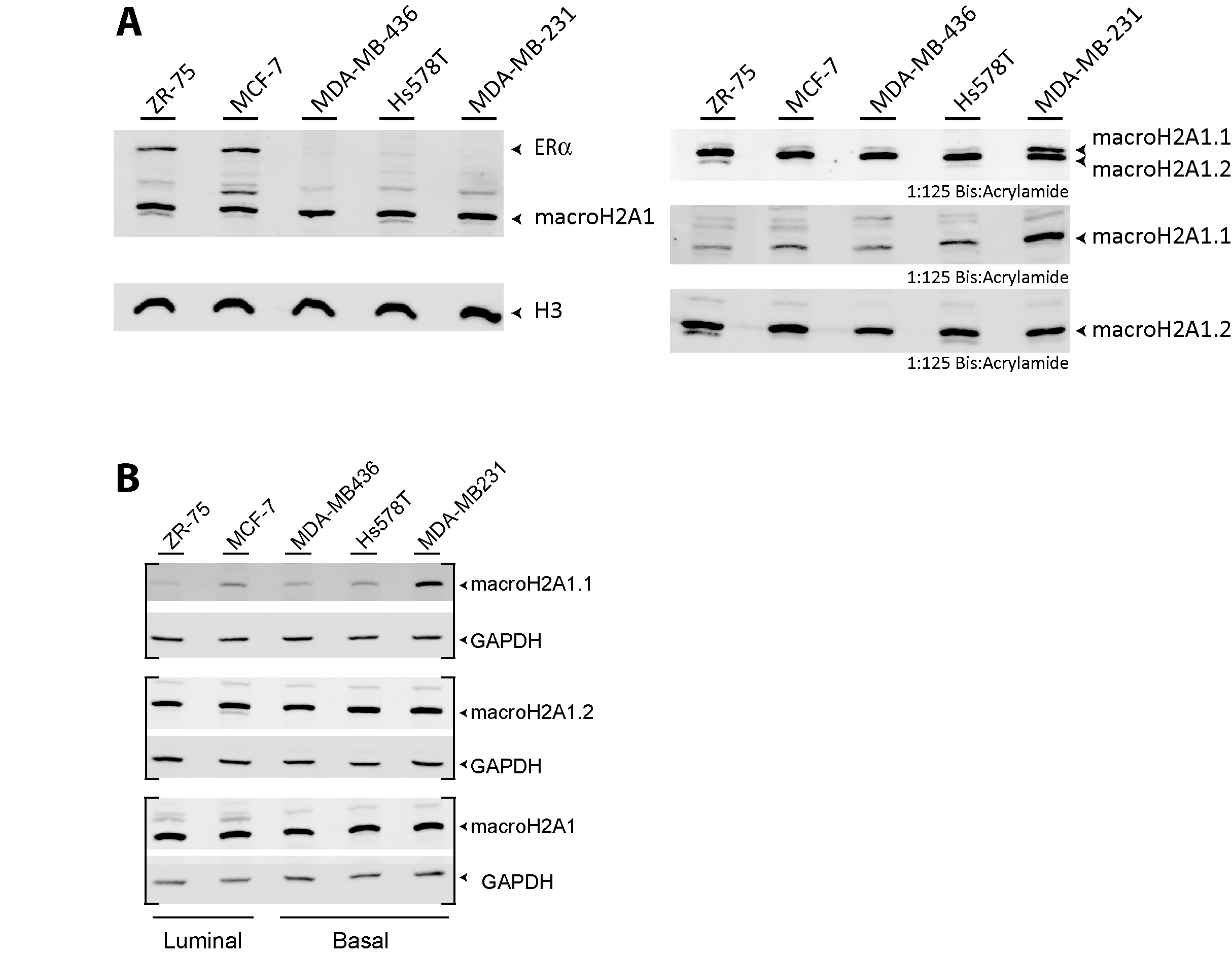

Supplement: Figure S1 — A- Characterization of α-macroH2A1 antibodies and cell lines. The specificity of α-macroH2A1 antibodies was verified using SDS-polyacrylamide gels low cross-linking (12.5% acrylamide, 1∶125 bisacrylamide) to separate the two splice variants macroH2A1.1 and macroH2A1.2. Total extracts of breast cancer cell lines were first resolved in SDS-polyacrylamide gels (standard (left) or low cross-linking (right panel)) then immunoblotted with α-macroH2A1 and α-ERα antibodies. Left panel: ERα, macroH2A1 and H3 specific antibodies. Right: top panel; macroH2A1 specific; middle panel: macroH2A1.1 specific; bottom panel: macroH2A1.2 specific antibody. B- MacroH2A1.1, macroH2A1.2, and total macroH2A1 protein expression levels in five breast cancer cell lines. Total protein extracts were immunoblotted with α-macroH2A1 (bottom panel), α-macroH2A1.1 (top panel) or α-macroH2A1.2 antibodies (middle panel). (TIF) [file pone.0098930.s001.tif]

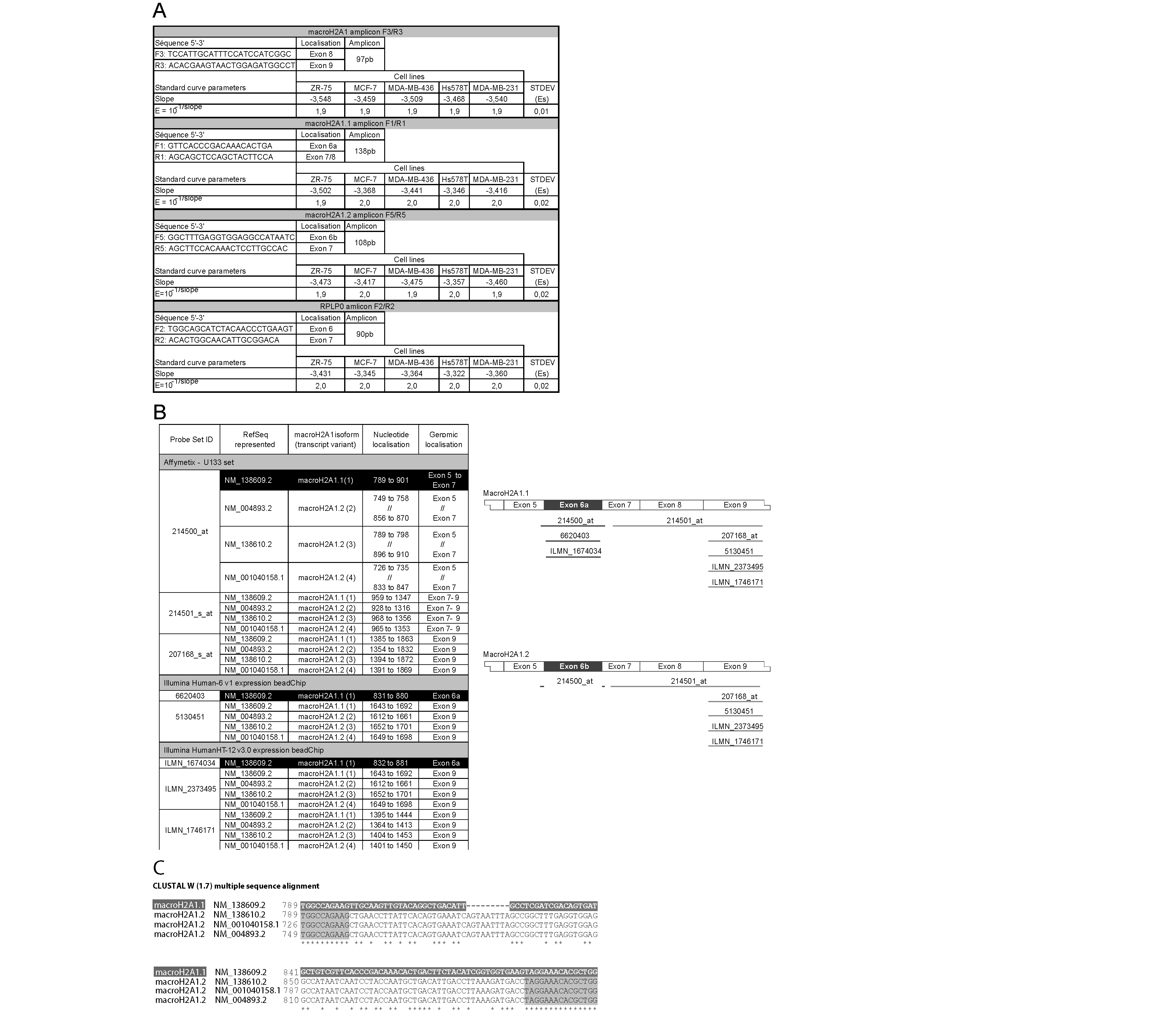

Supplement: Figure S2 — Primers, qPCR parameters and probe set IDs summary. A- Sequences, genomic location and size of amplicons generated by primers used in qPCR reactions are summarized. Parameters of the standard curve are reported for each pairs of primers in each cell lines used. For a given amplicon, efficiencies in the different cell lines are reported and compared each other (STDEV(Es)). B- Characterization of probe set ID of Affymetrix U133A, Illumina Human-6 v1 expression beadchip, IlluminaHuman HT-12 v3.0 expression beadchip arrays corresponding to macroH2A1 variants. For each probe, nucleotide reference sequences and macroH2A1 isoforms recognized are reported, as nucleotide and genomic localization of the sets of oligonucleotides presents at the probe ID. C- Clustal W multiple alignment of macroH2A1 variants sequences represented at the Probe Set ID 214500_at from U133A array. The sequences recognized by the probe are highlighted. (TIF) [file pone.0098930.s002.tif]

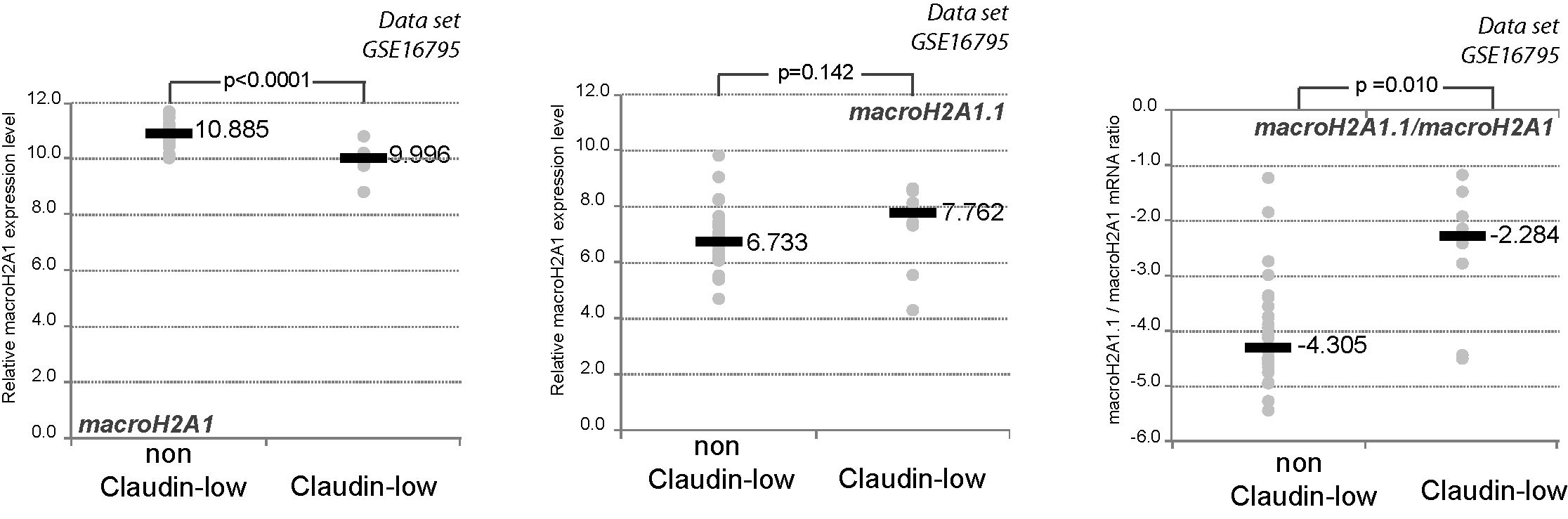

Supplement: Figure S3 — High macroH2A1.1 expression level in breast cancer cell lines characterizes Claudin-low molecular subtype. MacroH2A1.1/macroH2A1 mRNA ratios were determined for each cell line and classified according to molecular subtype assigned in the synthesis part of Table S1. In GSE16795 analysis [33], data from H3396 cell line are omitted as its subtype was not assigned. The median of macroH2A1.1/macroH2A1 values of each subgroup are specified. The reported p-values are the result of a two-tailed Mann-Whitney test. (TIF) [file pone.0098930.s003.tif]

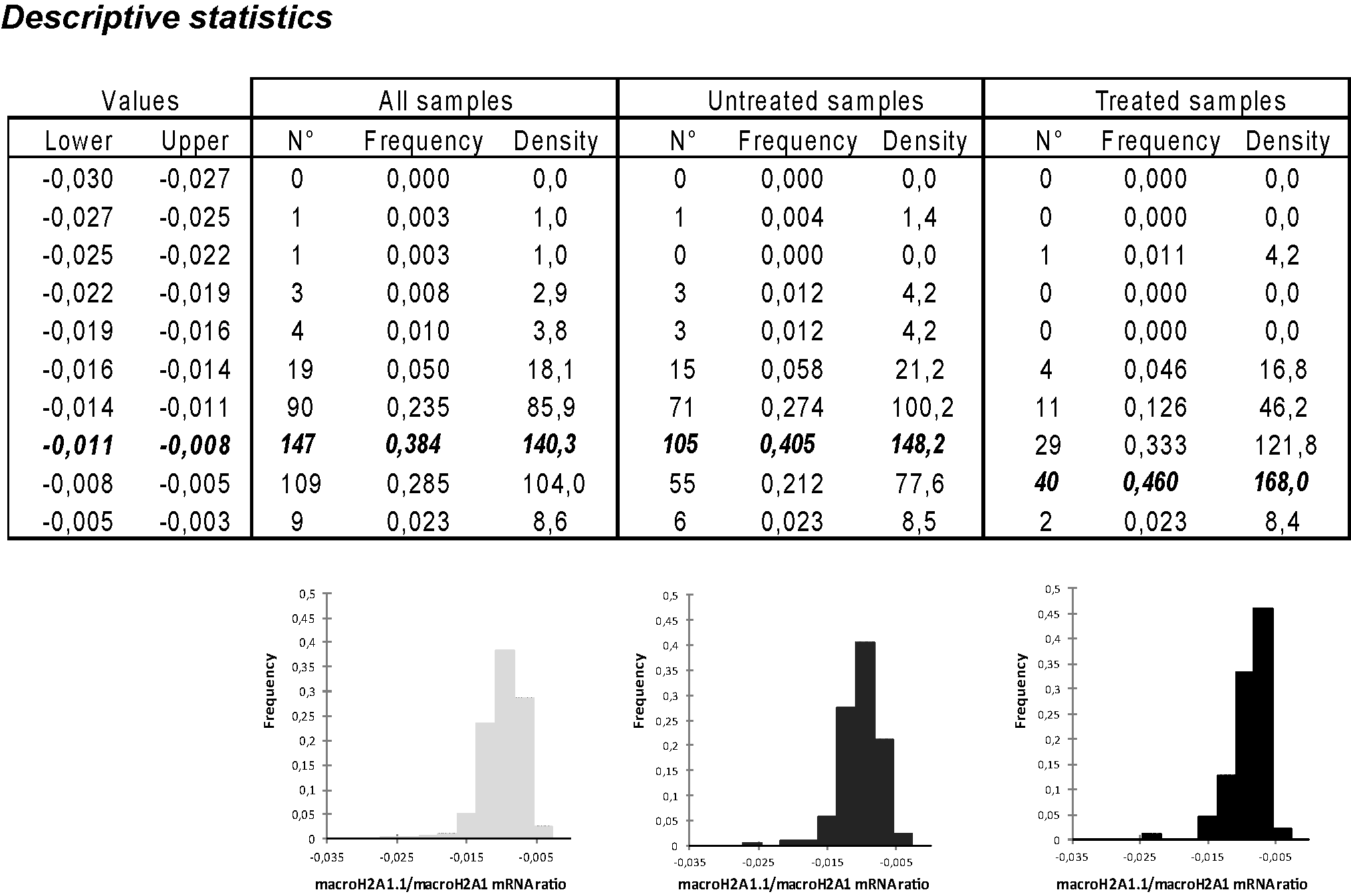

Supplement: Figure S4 — Analysis of the distribution of macroH2A1.1/macroH2A1 mRNA values in the different groups of patients studied. (TIF) [file pone.0098930.s004.tif]
