# Supplementary material for: Increased macroH2A1.1 Expression Correlates with Poor Survival of Triple-Negative Breast Cancer Patients
Source: PLoS One. 2014 Jun 9;9(6):e98930. doi: 10.1371/journal.pone.0098930 (PMC4049614; doi:10.1371/journal.pone.0098930)
Supplement: Table S1 — Summary of molecular subtype of breast cancer cell lines used in this studies. (PDF) [file pone.0098930.s005.pdf]

	E-TABM-157		E-MTAB-183	E-TABM-827 [1]	GSE16795	Miscellaneous	Synthesis	
**Reference**	**Neve et al., [2]**	**Prat et al., [3]**	**Lapuk et al., [4]**	**Synthesis**	**Hollestelle et al., [5]**	**References**		
**Cell line**								
**600MPE**	Luminal	Luminal	Luminal	-	Luminal		Luminal	
**AU565a**	Luminal	Luminal	-	-	-		Luminal	
**BT474**	Luminal	Luminal	Luminal	-	Luminal		Luminal	
**BT483**	Luminal	Luminal		-	Luminal		Luminal	
**CAMA1**	Luminal	Luminal	Luminal	Luminal	Luminal		Luminal	
**EVSA-T**	-	-	-	-	Luminal		Luminal	
**HCC1007**	Luminal	Luminal	-	-	-		Luminal	
**HCC1008**	*Unassigned*	-	-	-	-	Luminal - Haughian et al., [6]	Luminal	
**HCC1419**	-	-	-	Luminal	-	Luminal - Hoeflich et al., [7]	Luminal	
**HCC1428**	Luminal	Luminal	-	-	-		Luminal	
**HCC202**	Luminal	Luminal	-	-	-		Luminal	
**HCC2185**	Luminal	Luminal	-	-	-		Luminal	
**HCC2218**	-	-	-	-	-	Luminal - Hu et al., [8]	Luminal	
**IBEP1**	-	-	-	Luminal	-	Luminal - Buckley et al., [9]	Luminal	
**KPL1**	-	-	-	Luminal	-		Luminal	NON CLAUDIN-LOW
**LY2**	Luminal	Luminal	-	-	-		Luminal	
**MCF7**	Luminal	Luminal	Luminal	Luminal	Luminal		Luminal	
**MDAMB134VI**	Luminal	Luminal	-	Luminal	Luminal		Luminal	
**MDAMB175VII**	Luminal	Luminal	-	Luminal	Luminal		Luminal	
**MDAMB330**	-	-	-	-	Luminal		Luminal	
**MDAMB361**	Luminal	Luminal	Luminal	Luminal	Luminal		Luminal	
**MDAMB415**	Luminal	Luminal	Luminal	-	Luminal		Luminal	
**MDAMB453**	Luminal	Luminal	Luminal	Luminal	Luminal		Luminal	
**OCUB-F**	-	-	-	Luminal	Luminal		Luminal	
**SKBR3**	Luminal	Luminal	Luminal	Luminal	Luminal		Luminal	
**SK-BR-5**	-	-	-	-	Luminal		Luminal	
**SUM185PE**	Luminal	Luminal	Luminal	-	Luminal		Luminal	
**SUM44PE**	Luminal	Luminal	-	-	Luminal		Luminal	
**SUM52PE**	Luminal	Luminal	Luminal	Luminal	Luminal		Luminal	
**T47D**	Luminal	Luminal	Luminal	Luminal	Luminal		Luminal	
**UACC812**	Luminal	Luminal	-	-	Luminal		Luminal	

Data set	E-TABM-157		E-MTAB-183	E-TABM-827 [1]	GSE16795	Miscellaneous	Synthesis	
Reference	Neve et al., [2]	Prat et al., [3]	Lapuk et al., [4]	Synthesis	Hollestelle et al., [5]	References		
**Cell line**								
**UACC-893**	-	-	-	-	Luminal		Luminal	NON CLAUDIN-LOW
**ZR751**	Luminal	Luminal	Luminal	Luminal	Luminal		Luminal	
**ZR7530**	Luminal	Luminal	-	Luminal	Luminal		Luminal	
**ZR75B**	Luminal	Luminal	Luminal	-	-		Luminal	
N=	27	25	13	15	23			
**184A1N4**			Basal				Basal	NON CLAUDIN-LOW *(except for E-MTAB-183)
**184B5**			Basal				Basal	
**BT20**	Basal A	Basal A	Basal	BasalA	Basal		Basal A	
**CAL-51**	-	-	-	Basal B	-	Basal B - Sharpe et al., [10]	Basal B	
**DU4475**	*Unassigned*	-	-	Basal	*Unassigned*	Basal A - Kuemmerle et al.,[11]	Basal A	
**GI-101**	-	-	-	Basal	-	Basal - Mackay et al., [12]	Basal	
**HB4A**	-	-	-	Basal	-	Basal - Mackay et al., [12]	Basal	
**HCC1143**	Basal A	Basal A	Basal	Basal A	-		Basal A	
**HCC1187**	Basal A	Basal A	Basal	Basal A	-		Basal A	
**HCC1500**	Basal B	Basal B	Basal	Basal B	-		Basal B	
**HCC1569**	Basal A	Basal A	-	-	-		Basal A	
**HCC1599**	*Unassigned*	-	-	Basal A	-	Basal A - Hu et al.,[8]	Basal A	
**HCC1806**	-	-	-	Basal A	-	Basal A - Hu et al., [8]	Basal A	
**HCC1937**	Basal A	Basal A	-	BasalA	Basal		Basal A	
**HCC1954**	Basal A	Basal A	Basal	BasalA	-		Basal A	
**HCC2157**	Basal A	Basal A	-	-	-		Basal A	
**HCC3153**	Basal A	Basal A	Basal	-	-		Basal A	
**HCC38**	Basal B	Basal B	Claudin-low*	BasalB	-		Basal B	
**HCC70**	Basal A	Basal A	-	-	-		Basal A	
**MCF10A**	Basal B	Basal B	Basal	-	-		Basal B	
**MCF10F**			Basal				Basal	
**MCF12A**	Basal B	Basal B	Basal	-	-		Basal B	
**MDAMB468**	Basal A	Basal A	-	Basal A	Basal		Basal A	
**PMC42**	-	-	-	Basal	-	Basal - Mackay et al.,[12]	Basal	
**SK-BR-7**	-	-	-	Basal	Basal		Basal	
**SUM102PT**	-	-	-	-	Basal	Basal A - Heiser et al.,[13]	Basal A	

Data set	E-TABM-157		E-MTAB-183	E-TABM-827 [1]	GSE16795	Miscellaneous	Synthesis	
Reference	Neve et al., [2]	Prat et al., [3]	Lapuk et al., [4]	Synthesis	Hollestelle et al., [5]	References		
**Cell line**								
**SUM149PT**	Basal B	Basal B	-	Basal B	Basal		Basal B	NON CLAUDIN-LOW
**SUM190PT**	Basal A	Basal A	-	Basal A	-		Basal A	
**SUM225CWN**	Basal A	Basal A	-	-	-		Basal A	
**SUM229PE**	-	-	-	-	Basal	Basal - Mirzoeva et al.,[14]	Basal	
**VP229**	-	-	-	Basal	-	Basal - Brough et al.,[15]	Basal	
N=	**19**	**17**	**11**	**19**	**8**			
**BT549**	Basal B	Claudin-low	Claudin-low	Claudin-low	Basal		Claudin-low	CLAUDIN-LOW
**HBL100**	Basal B	Claudin-low	Claudin-low	-	-		Claudin-low	
**HCC1395**	-	-	-	Claudin-low	-	Claudin-low - Heiser et al.,[13]	Claudin-low	
**HS578T**	Basal B	Claudin-low	-	Claudin-low	Basal		Claudin-low	
**MDAMB157**	Basal B	Claudin-low	Claudin-low	Claudin-low	Basal		Claudin-low	
**MDAMB231**	Basal B	Claudin-low	Claudin-low	-	Basal		Claudin-low	
**MDAMB435s**	Basal B	Claudin-low	Claudin-low	Claudin-low	Basal		Claudin-low	
**MDAMB436**	Basal B	Claudin-low	-	Claudin-low	Basal		Claudin-low	
**SUM1315MO2**	Basal B	Claudin-low	-	-	Basal		Claudin-low	
**SUM159PT**	Basal B	Claudin-low	Claudin-low	Claudin-low	Basal		Claudin-low	
N=	**9**	**9**	**6**	**7**	**8**			

1. Holland DG, Burleigh A, Git A, Goldgraben MA, Perez-Mancera PA, et al. (2011) ZNF703 is a common Luminal B breast cancer oncogene that differentially regulates luminal and basal progenitors in human mammary epithelium. EMBO Mol Med 3: 167-180.
2. Neve RM, Chin K, Fridlyand J, Yeh J, Baehner FL, et al. (2006) A collection of breast cancer cell lines for the study of functionally distinct cancer subtypes. Cancer Cell 10: 515-527.
3. Prat A, Parker JS, Karginova O, Fan C, Livasy C, et al. (2010) Phenotypic and molecular characterization of the claudin-low intrinsic subtype of breast cancer. Breast Cancer Res 12: R68.
4. Lapuk A, Marr H, Jakkula L, Pedro H, Bhattacharya S, et al. (2010) Exon-level microarray analyses identify alternative splicing programs in breast cancer. Mol Cancer Res 8: 961-974.
5. Hollestelle A, Nagel JH, Smid M, Lam S, Elstrodt F, et al. (2010) Distinct gene mutation profiles among luminal-type and basal-type breast cancer cell lines. Breast Cancer Res Treat 121: 53-64.
6. Haughian JM, Pinto MP, Harrell JC, Bliesner BS, Joensuu KM, et al. (2012) Maintenance of hormone responsiveness in luminal breast cancers by suppression of Notch. Proc Natl Acad Sci U S A 109: 2742-2747.
7. Hoeflich KP, Merchant M, Orr C, Chan J, Den Otter D, et al. (2012) Intermittent administration of MEK inhibitor GDC-0973 plus PI3K inhibitor GDC-0941 triggers robust apoptosis and tumor growth inhibition. Cancer Res 72: 210-219.
8. Hu Z, Huang G, Sadanandam A, Gu S, Lenburg ME, et al. (2010) The expression level of HJURP has an independent prognostic impact and predicts the sensitivity to radiotherapy in breast cancer. Breast Cancer Res 12: R18.

9. Buckley NE, Conlon SJ, Jirstrom K, Kay EW, Crawford NT, et al. (2011) The DeltaNp63 proteins are key allies of BRCA1 in the prevention of basal-like breast cancer. Cancer Res 71: 1933-1944.
10. Sharpe R, Pearson A, Herrera-Abreu MT, Johnson D, Mackay A, et al. (2011) FGFR signaling promotes the growth of triple-negative and basal-like breast cancer cell lines both in vitro and in vivo. Clin Cancer Res 17: 5275-5286.
11. Kuemmerle NB, Rysman E, Lombardo PS, Flanagan AJ, Lipe BC, et al. (2011) Lipoprotein lipase links dietary fat to solid tumor cell proliferation. Mol Cancer Ther 10: 427-436.
12. Mackay A, Tamber N, Fenwick K, Iravani M, Grigoriadis A, et al. (2009) A high-resolution integrated analysis of genetic and expression profiles of breast cancer cell lines. Breast Cancer Res Treat 118: 481-498.
13. Heiser LM, Sadanandam A, Kuo WL, Benz SC, Goldstein TC, et al. (2012) Subtype and pathway specific responses to anticancer compounds in breast cancer. Proc Natl Acad Sci U S A 109: 2724-2729.
14. Mirzoeva OK, Das D, Heiser LM, Bhattacharya S, Siwak D, et al. (2009) Basal subtype and MAPK/ERK kinase (MEK)-phosphoinositide 3-kinase feedback signaling determine susceptibility of breast cancer cells to MEK inhibition. Cancer Res 69: 565-572.
15. Brough R, Frankum JR, Sims D, Mackay A, Mendes-Pereira AM, et al. (2011) Functional viability profiles of breast cancer. Cancer Discov 1: 260-273.
